# A Novel Strategy to Coat Dopamine-Functionalized Titanium Surfaces With Agarose-Based Hydrogels for the Controlled Release of Gentamicin

**DOI:** 10.3389/fcimb.2021.678081

**Published:** 2021-06-10

**Authors:** H. Melis Soylu, Pascale Chevallier, Francesco Copes, Federica Ponti, Gabriele Candiani, Fatma Yurt, Diego Mantovani

**Affiliations:** ^1^ Department Biomedical Technologies, The Institute of Natural and Applied Sciences, Ege University, Bornova, Turkey; ^2^ Laboratory for Biomaterials and Bioengineering, Canada Research Chair Tier 1, Department of Min-Met-Materials Eng., University Hospital Research Center, Regenerative Medicine, Laval University, Quebec City, QB, Canada; ^3^ GenT LΛB and µBioMI LΛB, Department of Chemistry, Materials and Chemical Engineering “G. Natta”, Politecnico di Milano, Milan, Italy; ^4^ Department Nuclear Applications, Institute Nuclear Science, Ege University, Bornova, Turkey

**Keywords:** spinal implants, Ti6Al4V, agarose, gentamicin, antibacterial coatings

## Abstract

**Introduction:**

The use of spinal implants for the treatment of back disorders is largely affected by the insurgence of infections at the implantation site. Antibacterial coatings have been proposed as a viable solution to limit such infections. However, despite being effective at short-term, conventional coatings lack the ability to prevent infections at medium and long-term. Hydrogel-based drug delivery systems may represent a solution controlling the release of the loaded antibacterial agents while improving cell integration. Agarose, in particular, is a biocompatible natural polysaccharide known to improve cell growth and already used in drug delivery system formulations. In this study, an agarose hydrogel-based coating has been developed for the controlled release of gentamicin (GS).

**Methods:**

Sand blasted Ti6Al4V discs were grafted with dopamine (DOPA) solution. After, GS loaded agarose hydrogels have been produced and additioned with tannic acid (TA) and calcium chloride (CaCl_2_) as crosslinkers. The different GS-loaded hydrogel formulations were deposited on Ti6Al4V-DOPA surfaces, and allowed to react under UV irradiation. Surface topography, wettability and composition have been analyzed with profilometry, static contact angle measurement, XPS and FTIR spectroscopy analyses. GS release was performed under pseudo-physiological conditions up to 28 days and the released GS was quantified using a specific ELISA test. The cytotoxicity of the produced coatings against human cells have been tested, along with their antibacterial activity against *S. aureus* bacteria.

**Results:**

A homogeneous coating was obtained with all the hydrogel formulations. Moreover, the coatings presented a hydrophilic behavior and micro-scale surface roughness. The addition of TA in the hydrogel formulations showed an increase in the release time compared to the normal GS-agarose hydrogels. Moreover, the GS released from these gels was able to significantly inhibit *S. aureus* growth compared to the GS-agarose hydrogels. The addition of CaCl_2_ to the gel formulation was able to significantly decrease cytotoxicity of the TA-modified hydrogels.

**Conclusions:**

Due to their surface properties, low cytotoxicity and high antibacterial effects, the hereby proposed gentamicin-loaded agarose-hydrogels provide new insight, and represent a promising approach for the surface modification of spinal implants, greatly impacting their application in the orthopedic surgical scenario.

## Introduction

Spinal implant surgery represents the most effective treatment for chronic back disorders such as spinal arthritis, scoliosis, vertebra fractures and others. Spinal implants are required to strengthen and improve the stability of the spine (Tomé-Bermejo et al., 2017), reducing pain and posture disorders. Spinal implants are generally made of titanium (Ti) or Ti-based alloys (Rao et al., 2014), such as Ti‐6AI‐4V. Ti alloys are used in virtue of their favorable properties such as lightweight, stress shielding, low density and biocompatibility. Moreover, Ti-based alloys exhibit excellent resistance to corrosion, and they have hydrophilic surface (Brunette et al., 2000), allowing proteins interaction and cells adhesion with the Ti surface. The main factor influencing the long-term success of spinal implants is their integration with bone, a process called osseointegration, and the absence of infection in the area surrounding the implants. Post-operative infections remain the main complication in implant-associated surgical procedures, affecting ~19% of patients undergoing these procedures (Schimmel et al., 2010; Quaile, 2012; Chahoud et al., 2014; Radcliff et al., 2015). Spinal implants wound infection treatments and re-hospitalization place an heavy burden on the healthcare system (Emerson et al., 2002). *Staphylococcus aureus* and *Staphylococcus epidermidis* remain the main bacteria involved in spinal implant infections (Gristina et al., 1989; Schierholz and Beuth, 2001; MacKintosh et al., 2006). Bacterial colonization inhibits the osseointegration process (Zaatreh et al., 2016), such that implant replacement is the most effective treatment. Unfortunately, additional surgery increases the chances of complications for the patients.

Biological responses are triggered by the implant surface properties. Therefore, Ti surface modifications and coatings have been investigated to prevent infections. The main strategies to obtain antibacterial effects rely on: contact-killing (e.g., chitosan coating), anti-adhesion (e.g., PEG) and antibacterial agent release (AARs; e.g., silver, copper, and antibiotic delivery system) (Orapiriyakul et al., 2018). For spinal implants applications, AARs are the most investigated due to the high versatility in terms of antibacterial agent and coating composition. All these approaches are summarized in **Table 1**. The advantage of AARs is the local delivery of antibacterial agents. Locally delivered antibiotics offer the advantage of a higher concentration at the wound site (Stall et al., 2009; Liu et al., 2016), as compared to systemic delivery. Among the different antibiotics, gentamicin sulfate (GS) is the most studied for this application (Eltorai et al., 2016). GS is an aminoglycoside antibiotic effective against both Gram positive and Gram negative bacteria (Singh et al., 2013). The main problem related to antibacterial coating for Ti implants is the short-lasting antimicrobial effects. In fact, the coatings described in **Table 1** are all characterized by rapid release of the loaded antibacterial agent. Thus, there is still problem in selecting a solution that efficiently addresses infection prevention at medium and long-term, i.e., more than one week and up to one month. In this light, the original idea explored in this work consists in developing a controlled-release system based on a biocompatible material, such as agarose, obtained by a process as simple as possible, and leading to an adherent coating. Agarose is a natural polysaccharide extracted from marine red algae (Anil et al., 2019). It has interesting properties for the proposed application, such as biocompatibility, e.g., non-immunological property, biomineralization, thermo-reversible gelation behavior, good mechanical strength and physicochemical features (Forget et al., 2013; Mizuta et al., 2013; Mishra and Kumar, 2014; Zarrintaj et al., 2018). In addition, agarose hydrogels ensure cell adhesion and provide adequate oxygen and nutrient permeability for cell growth (Zarrintaj et al., 2018). Besides, agarose is widely used as component in controlled drug delivery system (Meilander et al., 2003; Marras-Marquez et al., 2014; Nazemi et al., 2014; Awadhiya et al., 2016; Hu et al., 2016; Zarrintaj et al., 2018; Khodadadi Yazdi et al., 2020).

**Table 1:** Advantages and disadvantages of some coatings on Ti and Ti alloys, reported in the literature.

Coating composition *Coating process*	Advantages	Disadvantages	Ref.
Chitosan	- Drug release effective against *S. aureus.*	- 95% of vancomycin released after 5 min	(Swanson et al., 2011)
Vancomycin release *Dip coating*	- 65% of the drug released after 1 min - Total drug release 2.4 mg	- Multistep process (aminosilane, glutaraldehyde, chitosan grafting then chitosan/vancomycine coating) - Multilayer, thick and inhomogeneous coating	
Calcium alginate gelatin	- Excellent antibacterial activity against *S. aureus.*	- Antibacterial effect tests done after 18 hours	(Xiao et al., 2009)
Gentamicin release *Dip coating*	- During the first 8 h, 61.7% gentamicin released, then the release lasts for 10 days	- Multistep process - Unfriendly chemicals	
Polydopamine, hydroxyapatite (HAp), Ag NPs* and chitosan (CS)	- HAp/Ag/CS coating inhibited bacterial growth against *E. Coli* and *S. aureus*	- No indication of the antibacterial effect time - Long and multistep process	(Xie et al., 2019)
*Hybrid synthesis*	- No cytotoxicity	- Not reliable to a coating process	
HAp and AgNO_3_ *Plasma Electrolytic Oxidation treatment*	- After 20 h, silver ions significantly improved the antibacterial activity on *S. aureus* and *E. coli*	- Antibacterial activity effect for 1 day - Potential toxicity of Ag ions - Complex process	(Sobolev et al., 2019)
Dopamine, chitosan and immobilized RGD	- Adhesion of bacteria, *S. aureus* and *S, epidermis* decreased	- Bacteria adhesion tests done for only 4 h	(Shi et al., 2008)
RGD peptide release *Covalently grafted*	- Increase in osteoblast cell attachment and proliferation	- Unfriendly chemicals (HF, glutaraldehyde, etc.) - Multistep and long process (2 days)	
Thin films of either poly (2-hydroxyethyl methacrylate) or a copolymer based on poly (ethylene–glycol diacrylate) and acrylic acid Ciprofloxacin release *Electrosynthesis*	- Biocompatible - Antibacterial effect over 48 h on MRSA** - Burst release in first hour - After, low amounts of ciprofloxacin released over 1 week, less than the MIC***	- Short term release kinetics - Unfriendly chemical, e.g. monomers - Complex system as used a potentiostat-galvanostat system for the electrosynthesis	(De Giglio et al., 2011)

^*^NPs, nanoparticles; ^**^MRSA0, methicillin resistant Staphylococcus aureus (MRSA); ^***^MIC, minimum inhibition concentration.

To ensure its cohesion or to control its porosity, agarose is usually mixed with other components varying from surfactants (Tween 80, Pluronic F-68), natural polymers (chitosan, gelatin, fibrin, etc.), chemical crosslinkers (citric acid), or natural ones such as genipin and phenolic compounds (dopamine - DOPA, tannic acid - TA, caffeic acid, etc.) (Meena et al., 2007; Zhang et al., 2010; Marras-Marquez et al., 2014; Awadhiya et al., 2016; Zarrintaj et al., 2018). Phenolic compounds are of particular interest because they can act as crosslinker and also, as for TA, demonstrated to have antibacterial and anti-inflammatory properties of their own (Ninan et al., 2016; Glaive et al., 2017). Furthermore, polyphenol represents an environmentally friendly and economical approach to surface functionalization (Xu et al., 2016a). Indeed, this plant-derived phenolic compound rich in catechol moieties, e.g., dopamine, can form coatings on various substrates following polymerization under controlled conditions. Thus, functionalization of the Ti alloy surface through dopamine interface and the addition of TA in the agarose hydrogel formulation are supposed to have a beneficial impact on the agarose hydrogel cohesion and their attachment to the implant surface, ensuring its stability, and its biological properties.

Therefore, in this study, Ti alloy implant surfaces were grafted with a dopamine interface, coated with agarose-polyphenol based hydrogel, and further loaded with GS. This controlled release system was expected to be antibacterial up to one month, in order to prevent the formation of infection in the long term. In addition, calcium chloride was added to the hydrogel composition to investigate its effects on the gelation process. Moreover, it has the potential to induce the formation of hydroxyapatite and to enhance cell adhesion (Elshahawy et al., 2014; Haraguchi et al., 2020). Ultraviolet (UV) lighting was used as sterilization method and crosslinking activator. The modified surfaces were characterized in order to validate the coating deposition, composition, homogeneity and biocompatibility. Finally, the amount of released antibiotic and its effects on bacteria was investigated as well.

## Materials and Methods

All reagents used were of analytical grade without further purification. Agarose, TA, DOPA hydrochloride, tris (hydroxymethyl) aminomethane (Tris), and calcium chloride (CaCl_2_) were acquired from Sigma-Aldrich (Oakville, ON, Canada). GS was purchased from Sigma-Aldrich (G1914; Oakville, ON, Canada). Acetone, methanol, sodium hydroxide (NaOH) were purchased from Laboratoire MAT (Québec, Qc, Canada).

### Surface Modification

The metallic surface modification was addressed in four steps: (i) sample cleaning, (ii) surface activation by sodium hydroxide (NaOH), (iii) DOPA grafting, and (iv) agarose coating and antibiotic loading. Samples have been cleaned to remove any contaminants that could alter the grafting procedure, while NaOH activation aims to produce OH-groups on the Ti surface to improve the grafting of the DOPA molecules.

#### Preparation of Ti6Al4V Samples

Sandblasted Ti6Al4V-ELI discs of both 14- and 16-mm diameters and 3 mm in thickness were kindly supplied by METROSAN (Manisa, Turkey). These samples were cleaned in an ultrasonic bath for 10 min in each of the following solutions: acetone, deionized water, and methanol. After cleaning, samples were dried with compressed air and stored under vacuum until further use.

#### Surface Pre-Treatment

After the cleaning process, surface pre-treatments were performed at three different molarities (i.e., 2.5, 4 and 6 M) of NaOH aqueous solutions in order to identify the best activation condition to be used for enhancing the coating grafting. This activation was performed by immersing the cleaned samples in the NaOH solutions for 15 min in an ultrasonic bath. Then, the samples were thoroughly rinsed with deionized water and dried with compressed air.

#### Dopamine Grafting

Ti6Al4V activated samples were placed into a 12-well plate and 3 mL of a 2 mg/mL DOPA solution were added to each sample, and allowed to react for 24 hours (Campelo et al., 2017). To prepare the solution, DOPA was dissolved in 10 mM Tris buffer and the pH of this solution was adjusted to 8.7 using a 2 M NaOH aqueous solution. After DOPA grafting, the samples were washed thoroughly with deionized water before further grafting. The DOPA-grafted samples are hereafter referred to as C1 samples.

#### Antibiotic Loading on Agarose Hydrogel and Coating on Metallic Surfaces

Four different conditions have been investigated in order to compare the chemical and biological effects of each component, for instance the effect of TA or CaCl_2_ on the antibiotic release kinetics. The conditions are the following: 1) Agarose (C2), 2) Agarose-GS (C3), 3) Agarose-GS-TA (C4) and 4) Agarose-GS-TA-CaCl_2_ (C5).

The solutions used to obtain the different coatings were prepared as follow: aqueous agarose solution at 1% (w/v) prepared under agitation at 80°C; GS aqueous solution at 1 mg/mL. For C4 and C5 conditions, TA and CaCl_2_ were directly added in the GS solution, to reach a final concentration of 0.1% (w/v). All the solutions were kept in a water bath at 60°C before use to avoid agarose gelation.

The as-prepared solutions were poured onto the dopamine grafted Ti discs using a micropipette in order to fully cover the surface (200 µL). Then, the samples were placed in a UV oven (MBI Lab Equipment, Montreal, Canada) and irradiated with UV light at λ = 254 nm for 2 hours with an intensity of 2.7 mW/cm. As a result of the UV irradiation, the crosslinks in the hydrogel structure were strengthened and, in addition, the sterilization of the samples was ensured. **Table 2** briefly summarizes the different experimental conditions used in the study.

**Table 2:** Different coating conditions of Ti6Al4V surfaces.

Conditions	
**C1**	Ti6Al4V-DOPA
**C2**	Ti6Al4V -DOPA-Agr
**C3**	Ti6Al4V -DOPA-Agr-GS
**C4**	Ti6Al4V -DOPA-Agr-GS-TA
**C5**	Ti6Al4V -DOPA-Agr-GS-TA-CaCl_2_

### Surface Characterization

After each modification step, the surface morphology, wettability as well as composition were assessed by profilometry analyses, contact angle measurements, X-ray photoelectron spectroscopy (XPS) and Fourier-Transform Infrared (FTIR), respectively.

The surface roughness of crude and modified samples was measured by surface profilometer measurements on a scan area of 1 mm x 1 mm using a Bruker Dektak XT Profilometer (MA, USA) with a tip radius of 12.5 µm and a stylus force of 3 mg.

Surface wettability was investigated on sandblasted, pre-treated and grafted samples *via* static and dynamic contact angle measurements, which were recorded using a VCA 2500 XE system (AST^®^ Billeria, MA, USA). The analyses were performed at room temperature, with 1 μL ultrapure water droplets applied in three different regions per sample.

Surfaces atomic compositions were detected by XPS analyses using a Physical Electronics PHI 5600-ci equipment (MN, USA). A standard aluminum X- ray source (1486.6 eV) was used to record survey spectra, while high-resolution spectra were recorded with a standard magnesium X-ray source (1253.6 eV). All spectra were recorded without charge compensation. Detection was carried out at a 45° angle with respect to the surface normal, and the analyzed area was 0.5 mm^2^. The curve fitting procedure of the components underlying the C1s peaks was performed by means of a least-square minimization procedure employing Gaussian–Lorentzian functions and a Shirley type background. The C1s peaks were referenced at 285 eV (C-C and C-H).

The surface chemical changes were also investigated by Attenuated Total Reflectance-Fourier Transformed Infrared (ATR-FTIR) spectroscopy using a commercial spectrometer (Agilent Cary 660 FTIR, Agilent Technologies, CA, USA), equipped with a deuterated L-Alanine-doped triglycine sulfate (DLa-TGS) detector and a Ge-coated KBr beam splitter. Spectra were recorded in absorbance mode and 64 scans were recorded between 500 cm^−1^ to 4,000 cm^−1^ with a spectral resolution of 4 cm^-1^.

### Antibiotic Release Study

The agarose gel-coated Ti samples (C2, C3, C4 and C5), prepared as previously described, were placed in 12-well plates and 2 mL of McCOY’s 5A medium (Biological Industries), were added in each well. The plates were incubated at 37°C with 5% CO_2_ and 95% humidity. At established time points (i.e., 6 hours, 1, 3, 7, 14, 21 and 28 days) the solution covering the samples was completely removed and replaced with the same volume of fresh medium. The eluates were stored until day 28. Next, GS release from samples was quantified using a specific enzyme-linked immunosorbent assay (ELISA, Creative Diagnostics), according to the manufacturer’s instructions.

### Biological Assays: Cell Culture

#### Cell Culture

Human sarcoma osteogenic (Saos-2, ATCC HTB-85) cells were cultured in McCoy’s 5A medium supplemented with 15% (v/v) fetal bovine serum (FBS), 1% L-glutamine, penicillin (100 U/mL) and streptomycin (100 U/mL). Cells were maintained at 37°C in a saturated atmosphere at 5% CO_2_ (hereafter referred to as standard culture conditions). Culture media was changed every two days until a 90% of confluence was reached. Then, cells were enzymatically detached from the plate then re-plated at a ratio of 1:5. Cells at passage 5 and 6 were used for the experiments.

#### Indirect Cytotoxicity Test

Indirect cytotoxicity tests were performed according to ISO 10993–5 to evaluate whether toxic products were released. Ti coated samples (disk of 14 mm diameter, 1.53 cm^2^) were incubated in 2 mL of culture medium at 37°C in a saturated atmosphere at 5% CO_2_. After 6 hours, 1, 3 and 7 days, the medium was collected and stored until testing.

Saos-2 cells were seeded in a 96-well plate (n= 5 well/condition) at a density of 2x10^4^ cells/cm^2^. After 24 h, culture medium was removed and replaced with 100 µL of the previously collected eluates or complete medium (i.e., Control). Cell viability was evaluated after 4h of incubation with an MTT assay solution (Amresco), according to the manufacturer’s guidelines. Briefly, the culture medium was replaced with 100 µL of culture medium containing 10% of MTT compound. Samples were incubated for 4 hours in standard culture conditions. After the incubation, the formazan product obtained by the reduction of MTT reagent by means of the mitochondrial activity was solubilized using 100 µL/well of dimethyl sulfoxide (DMSO) (Sigma Aldrich). The obtained solutions were then transferred to a new 96-well plate and the absorbance, at λ = 570 nm, was measured with a multi-plate reader (Varioskan Flash Multimode Reader Thermo, Finland). The obtained results were normalized over the Control condition (100%).

### Zone of Inhibition (ZOI) Test

A *S. aureus* (DSM 346, Gram positive bacterium, Class 2 biological agent) suspension, at a bacteria concentration of 1x10^8^ colony forming units (CFU)/mL, was homogenously distributed onto Mueller-Hinton agar-coated petri dishes and then incubated for 24 hours in the oven at 37°C. Afterward, Ti6Al4V disc samples with different coating compositions (C1, C2, C3, C4 and C5) were placed on the prepared plates (n = 3). In addition, as a standard of GS, a sterile cellulose filter with a diameter of 6 mm was loaded with GS at a concentration of 1 mg/mL and placed on the petri dish like the tested Ti6Al4V discs. The petri dishes were then stored in an incubator at 37°C for 24 hours. The diameters of the growth inhibition zones formed around the discs after 24 hours were measured with a caliper (Rao et al., 2014). To evaluate the antibacterial efficiency of the tested coatings, the efficiency ratio was calculated as follows:

$$Efficiency\;Ratio=\frac{Inhibition\;zone\;Surface}{Sample\;Surface}$$

### Antibacterial Activity of Hydrogels-Released Gentamicin

Assessments of the antibacterial activity of the released GS were performed on the following materials: C1; C2; C3; C4 and C5. For sterilization, samples (n = 3 samples/condition) were UV-irradiated for 30 min per side. Briefly, samples were placed in 6-well plate and 2 mL of sterile nutrient broth (NB, containing 3 g/L of meat extract, 10 g/L of casein peptone and 15 g/L of sodium chloride) were added to each sample-containing well. Samples were next incubated under stirring at 37°C in a humidified atmosphere. After 6 hours, 1, 3 and 7 days, eluates were collected and replaced with 2 mL of fresh sterile NB. All the eluates were kept at -20°C until further testing.

*In vitro* susceptibility tests were performed against *S. aureus* using a protocol derived from the UNI EN ISO 20776-1 norm. Briefly, bacteria were pre-cultured in 5 mL of NB at 37°C under shaking at 130 rpm for 18 hours in order to reach an optical density at λ = 600 nm (i.e., OD_600nm_) of ≈1, corresponding to 1x10^9^ CFU/mL. The bacterial suspension was then serially diluted in NB broth to adjust the final concentration to 1x10^6^ CFU/mL. Afterwards, 50 μL/well of such inoculum was plated in a 96-well plate, and 50 μL/well of each eluate (n = 3 per sample) was added in each well. Bacteria grown with 100 μL/well of NB broth were used as positive growth controls, while wells containing 100 μL of antibiotic-free NB broth were used as negative controls (Bono et al., 2019). After 24-hrs incubation at 37°C, the OD_600nm_ of each well was read by means of a multiplate reader (GENios Plus reader, Tecan, Italy). The antimicrobial activity of the released GS from antibiotic-loaded coatings at each time point, with respect to the C1 condition, was expressed as the percentage of bacterial survival (%) as follows:

$$Bacterial\;Survival(\%)=\frac{OD600nm\;Sample}{OD600nm\;C1}\;\times 100$$

The minimum inhibitory concentration (MIC) of GS against *S. aureus* was assessed as well according to UNI EN ISO 20776-1 norm (British Standards Institution, 2006) and used as internal reference. Briefly, GS stock solution at 10 mg/mL was serially diluted in sterile NB broth to give final concentrations ranging from 1 mg/mL to 0.25 μg/mL. Afterwards, each GS solution was mixed with 50 μL/well of the test inoculum and the antibacterial activity was calculated as described herein above.

### Statistical Analysis

The statistical analysis of the samples was calculated using a Kruskal-Wallis multiple comparisons test and nonparametric ANOVAs with InStatTM software (GraphPad Software, La Jolla, CA, USA). All data reported are means ± SD of at least three independent experiments. The confidential range selected was 95%, which gives statistical differences when P-values < 0.05.

## Results

### Surface Pretreatment and Dopamine Grafting

#### Surface Pretreatment Optimization

Before DOPA grafting, Ti6Al4V samples were pre-treated with 2.5 M, 4 M and 6 M NaOH solutions to generate hydroxyl groups on the surface. The presence of hydroxyl groups on the surface was evaluated by static contact angle measurement. Indeed, an increase in surface polarity should increase the wettability. The results of contact angle values exhibited a major difference between pre-treated surfaces and crude Ti6Al4V: from 28.7 ± 0.3° for crude sample to 6.6 ± 0.6°, 5.1 ± 0.2° and 4.8 ± 0.7° for samples treated with 2.5 M, 4 M and 6 M NaOH solutions, respectively. The pretreated samples morphology was also evaluated by profilometry measurements. The average roughness (Ra) values for NaOH-pretreated samples exhibited a higher roughness compared to sandblasted sample, ranging from 0.55 ± 0.09 µm for crude sample to 0.58 ± 0.01 µm, 0.98 ± 0.62 µm, 1.08 ± 0.39 µm for samples treated with 2.5 M, 4 M and 6 M NaOH, respectively. Furthermore, higher NaOH concentrations led to a significant increase in roughness, meaning that the basic medium attack appears to be more aggressive. The molarity of NaOH did not exhibit any effect on the contact angle values, whereas it has a significant one on the roughness. As higher roughness is known to favor bacteria adhesion, 2.5 M NaOH has been chosen for further experimentation.

#### Dopamine Grafting on Pretreated Samples

The impact of DOPA-grafting on the surface hydrophilicity and morphology was evaluated by static contact angle measurements and profilometry (**Table 3**). The static contact angle value of DOPA-grafted sample increased when compared to that of pretreated Ti6Al4V sample: from 66.8 ± 2.6° to 6.6 ± 0.7°, respectively. Even though the contact angle value increased, the modified surfaces remained hydrophilic, since the contact angle value remained below 90°. Also, the average roughness, Ra, of DOPA-coated sample is similar to that of the sandblasted and pretreated sample (**Table 3**), meaning that the polydopamine layer is homogeneous. The dopamine-grafting efficiency was further assessed by XPS analyses allowing to determine the atomic surface composition (**Table 3**). After DOPA grafting, surface composition drastically changed compared to the pretreated one. Indeed, a significant increase of C amount, decrease of O percentage and 8.2 ± 0.9% of nitrogen were detected, whereas no nitrogen was noticed on the pretreated sample (**Table 3**). In addition, the relative percentage of C, O and N are close to those expected based on dopamine structure. Besides, it should be underlined that, on DOPA grafted surface, no Ti was detected (**Table 3**), meaning that the coating thickness is higher than 5 nm (XPS depth analysis). In addition, this evidences that the surface was homogeneously coated with DOPA, as earlier deduced from the roughness value.

**Table 3:** Average XPS atomic surface composition, surface roughness and contact angle values for crude Ti6Al4V, pretreated and DOPA grafted samples.

Sample	Atomic Percentage %				Ra(µm)	CA (°)
	C	O	N	Ti		
Ti6Al4V	27.4 ± 2.1	52.9 ± 1.3	–	12.2 ± 0.8	0.55 ± 0.09	28.7 ± 0.3
Pretreated	24.0 ± 1.6	55.5 ± 0.9	–	20.5 ± 0.8	0.58 ± 0.01	6.6 ± 0.7
DOPA	70.6 ± 0.8	21.2 ± 0.9	8.2 ± 0.9	–	0.59 ± 0.03	66.8 ± 2.6

### Antibiotic Loading on Agarose Hydrogel and Coating on Metal Surfaces

On the dopamine-grafted surfaces (C1), the coating step was then carried out. As previously mentioned, several agarose-based coatings, named C2 to C5 (**Table 2**), were prepared. The effect of each component, namely GS, TA and CaCl_2_, on coating properties, antibiotic release profile, and cell viability was evaluated. Besides, the homogeneity, composition, hydrophilicity and stability of the as-obtained coatings were assessed as well.

**Figure 1:**
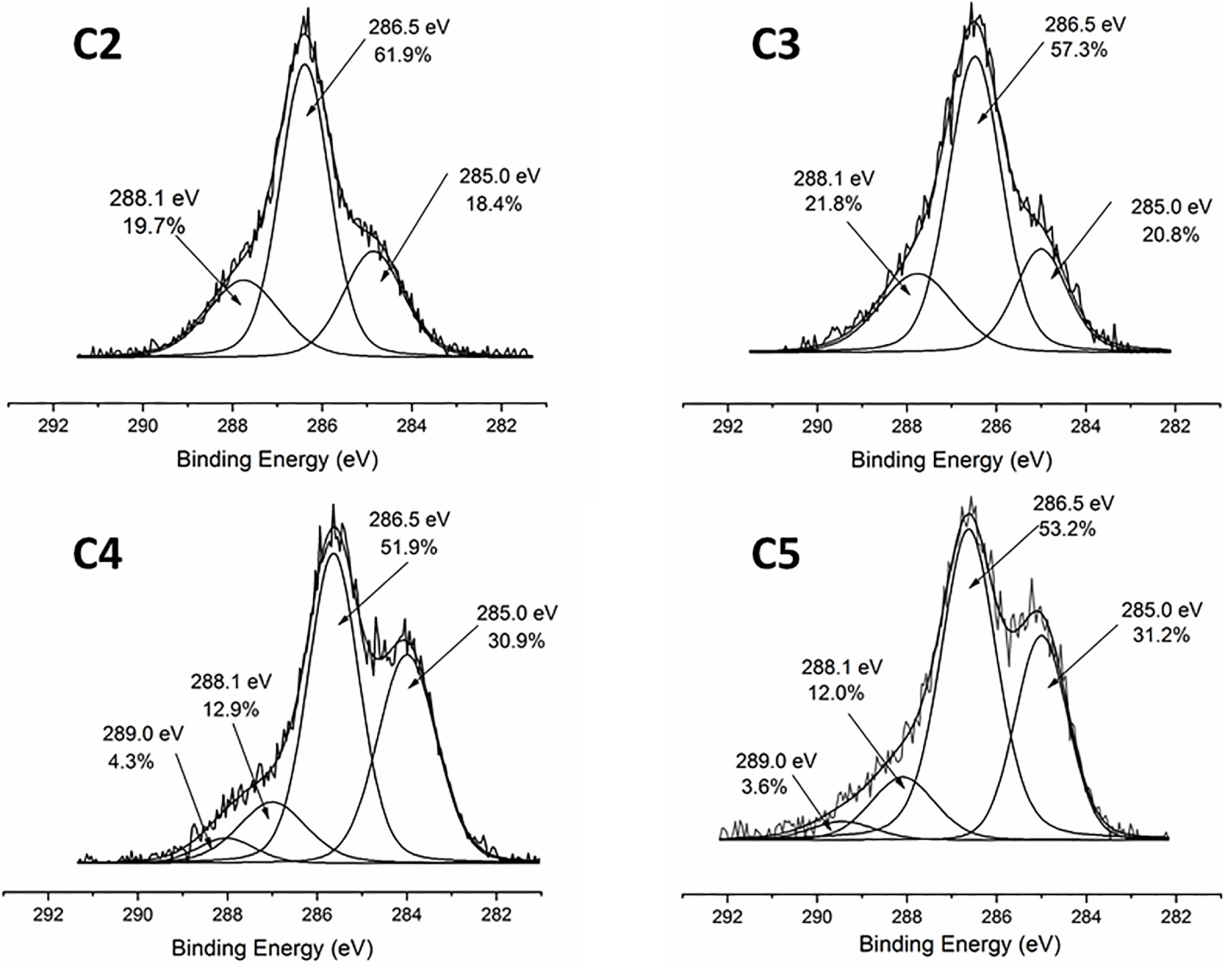
XPS high resolution C1s spectra of agarose-based coating on Ti6Al4V surfaces: C2-Agr; C3-Agr/GS; C4-Agr/TA/GS, and C5-Agr/TA/GS/CaCl_2_.

**Figure 2:**
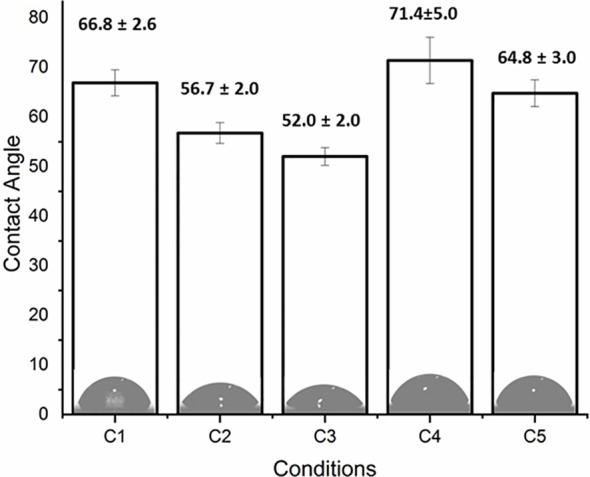
Contact angle measurements and water drop images on the sample’s surfaces.

**Figure 3:**
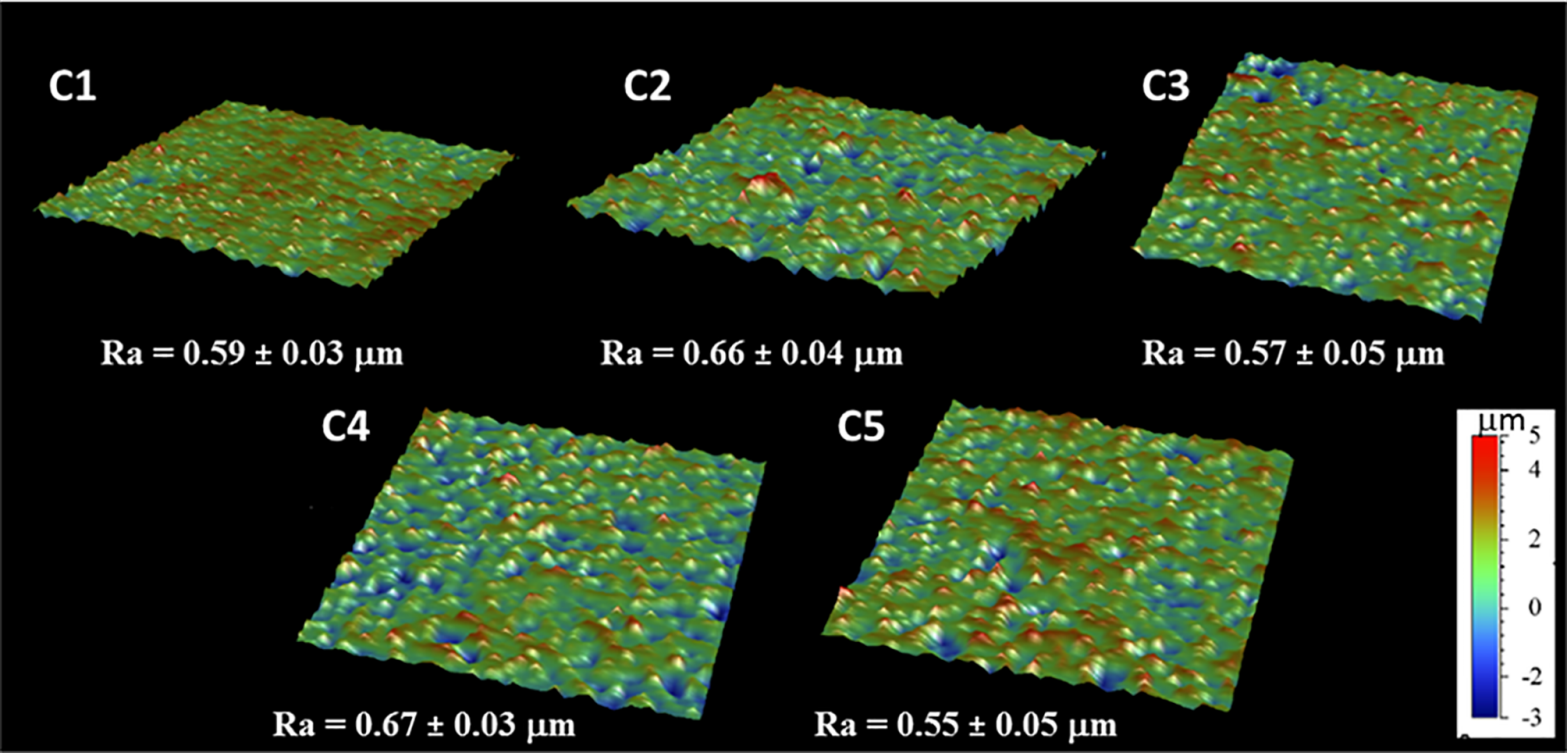
Topographic maps for Ti6Al4V-DOPA and the different agarose-based coatings.

The coating efficiency is clearly evidenced by the surface composition assessed by XPS survey analyses (data shown in **Table 4**). Indeed, there is a huge increase of oxygen due to the presence of agarose. In addition, the nitrogen initially detected on C1, associated to DOPA structure, is only at trace level (< 1%) on agarose-based samples (C2, C3, C4 and C5). This means that the coatings are relatively homogeneous and that the agarose-based coating is approximately 5 nm thick (XPS depth analysis). Despite small variations, the surface composition of the different coatings appears similar, around 62% of carbon and 36% of oxygen, with relative percentages close to the agarose structure. Therefore, it appears that the different components in the coatings do not change the surface composition, even though small contributions of Ca and Cl, with a ratio 1 to 2, were detected in sample C5 (presence of CaCl_2_). Surprisingly, GS was not detected even if this molecule contains in its structure specific atoms, such as sulfur and nitrogen, thus suggesting that GS resulted more trapped in the coating than on the first layers of the surface. The occurrence of TA, composed only of carbon and oxygen, was not evidenced in XPS spectra. On the other hand, C1s high-resolution spectra clearly demonstrate the presence of TA (**Figure 1**). Indeed, the TA contained in C4 and C5 samples led to an increase of C-C/C-H peak at 285 eV compared to C3, as in its structure TA has many aromatic rings. In addition, TA contains several O-C=O moieties, which are detected at 289.0 eV. This peak was not observed before on C2 and C3 coatings. The other C1s high-resolution peaks at 286.5 eV and 288.1 eV are associated to C-O bonds, coming from the alcohol groups and O-C-O from the acetal moieties, respectively. These groups are characteristic of glycoside derivatives such as agarose and GS. Regarding the effect of the coating compositions on the wettability, the surface remains hydrophilic (contact angle value less than 90°; **Figure 2**). There is a slight decrease of contact values from DOPA to DOPA-Agarose-GS, from 68° to 52°, associated to the hydroxyl groups of glycoside structure. On the other side, C4 and C5, both containing TA in their composition, exhibit higher water contact angle values, up to 70°. This can be associated to the composition of TA, which is known to be a relatively hydrophobic molecule due to the presence of aromatic rings. Furthermore, their contributions have been previously noticed in high-resolution C1s spectra (**Figure 1**). As previously stated, the roughness plays a critical role in terms of protein adsorption and cell adhesion. Therefore, the surface roughness of coated samples was also evaluated by profilometry measurements. Ra values are given in **Figure 3**. The images, as well as Ra values, show that the grafting does not induce significant changes in terms of sample morphology (**Figure 3**), Moreover, the Ti6Al4V-DOPA and the agarose-based coatings, whatever their composition, appear homogeneous and the roughness remain at the micrometer level scale.

**Table 4:** Average XPS atomic composition of the different coatings.

Sample	Atomic percentage				
	% C	% O	% N	% Ca	% Cl
**C1**	70.6 ± 0.8	21.2 ± 0.9	8.2 ± 0.9	–	–
**C2**	62.3 ± 0.4	37.0 ± 0.2	0.7 ± 0.4	–	–
**C3**	61.3 ± 1.0	38.4 ± 0.7	0.3 ± 0.5	–	–
**C4**	63.9 ± 1.1	35.5 ± 1.6	0.6 ± 0.5	–	–
**C5**	63.4 ± 0.7	34.1 ± 0.9	–	0.9 ± 0.4	1.6 ± 1.1

### Antibiotic Release Study

**Figure 4:**
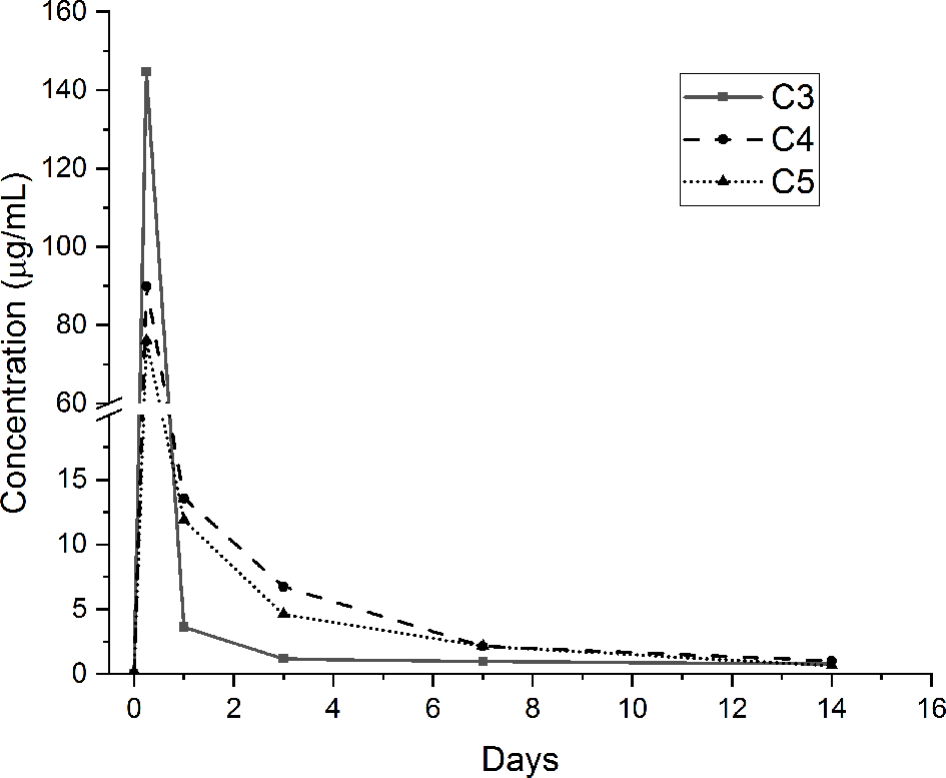
Gentamicin (GS) release concentrations from GS-loaded agarose-coated samples.

The release of GS from the agarose hydrogel coatings containing the loaded antibacterial compound (i.e., C3, C4 and C5 conditions) was performed in pseudo-physiological conditions for up to 28 days. GS amount has been determined using a GS-specific ELISA quantification assay. GS release curves for the studied periods are presented in **Figure 4**. As shown, it can be noticed that the release of GS from the tested samples is characterized by a burst peak at 6 hours (C3: 144.6 ± 49.1 µg/mL; C4: 89.9 ± 14.6 µg/mL; C5: 76.1 ± 15.7 µg/mL). The amount of GS released at this time point by the C3 condition is significantly higher than the C5 condition (p <0.05). After 1 day, the amount of released GS from the C3 samples is drastically reduced (3.6 ± 1.4 µg/mL) and significantly lower compared to the C4 (13.6 ± 0.5 µg/mL; p <0.001) and C5 (11.9 ± 2.6 µg/mL; p <0.001) conditions. After 3 days, the C4 (6.7 ± 0.6 µg/mL) and C5 (4.6 ± 0.1 µg/mL) samples released significantly higher concentrations of GS compared to the C3 condition (1.2 ± 0.2 µg/mL; p <0.001 *vs.* both C4 and C5). After 7 days, once again, the concentration of the released GS resulted to be significantly higher for the C4 (2.1 ± 0.4 µg/mL) and C5 (2.1 ± 0.2 µg/mL) conditions compared to the C3 one (1 ± 0.1 µg/mL, p <0.001 *vs.* both C4 and C5). Finally, after 14 days of incubation, the amount of GS released by the three coatings was drastically reduced (below 1 µg/mL) and no significant differences were noted between the tested conditions. After 21 and 28 days of incubation, all the tested conditions released negligible amounts of GS (≃ 0.1 µg/mL, data not shown). Of note, the FTIR analysis performed after 28 days of incubation showed that the agarose-based coating, C2, C3, C4 and C5, displayed the same chemical composition they had at the beginning of the test, thus demonstrating the stability of the applied coating (data in **SI –** **Figure S1**).

### Indirect Cytotoxicity Results

**Figure 5:**
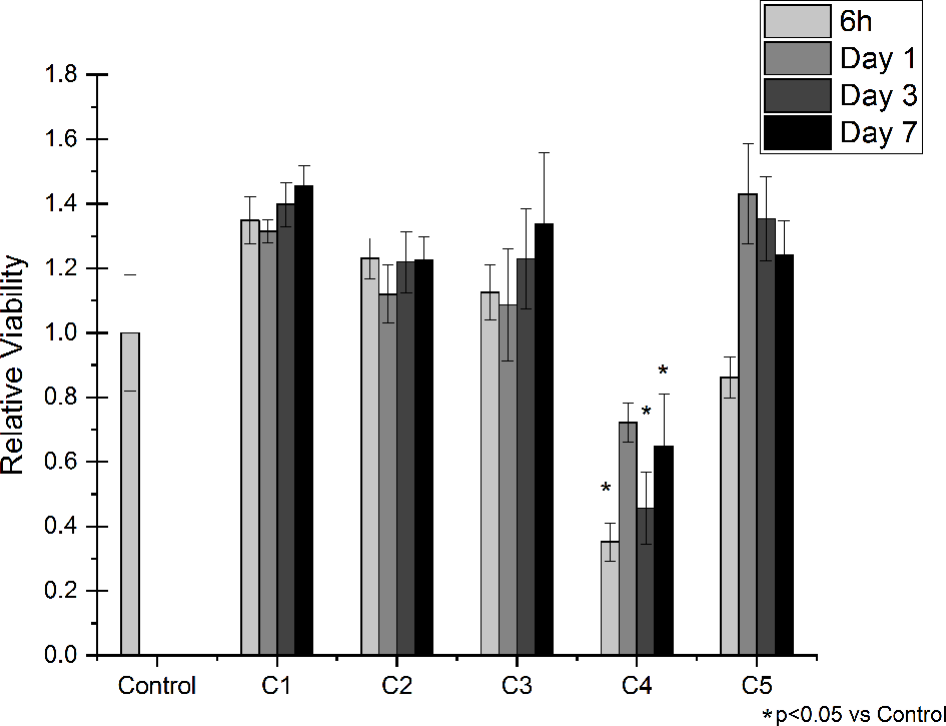
*Indirect Viability Assay*. Saos-2 cells were treated with conditioned medium collected after 6 hours, 1, 3 and 7 days of incubation with the following conditions: C1, C2, C3, C4 and C5. Normal culture media was used as a control (Control). The graphic shows the relative viability ± SD for each condition.

Indirect viability tests were performed on Saos-2 cells to evaluate if the released medium obtained from the different agarose gel-based coatings had cytotoxic effects. Indirect cell viability results are shown in **Figure 5**. Of note, eluates from the C1 and C2 did not affect cell viability for all the tested time points, meaning that the DOPA-functionalization and the agarose gel by themselves do not exert cytotoxic effects. Similarly, the released medium obtained from C3 samples did not show any negative effect on the Saos-2 viability, meaning that the released GS does not have cytotoxic effects on cells. However, in presence of the medium obtained by the C4 samples, cell viability at all the tested time points resulted being significantly reduced compared to the Control condition. Interestingly, the addition of CaCl_2_ (i.e., C5 sample) resulted being effective in increasing cell viability compared to C4, showing results comparable to the one obtained in presence of the C1, C2 and C3 medium.

### Antibacterial Test Results

**Figure 6:**
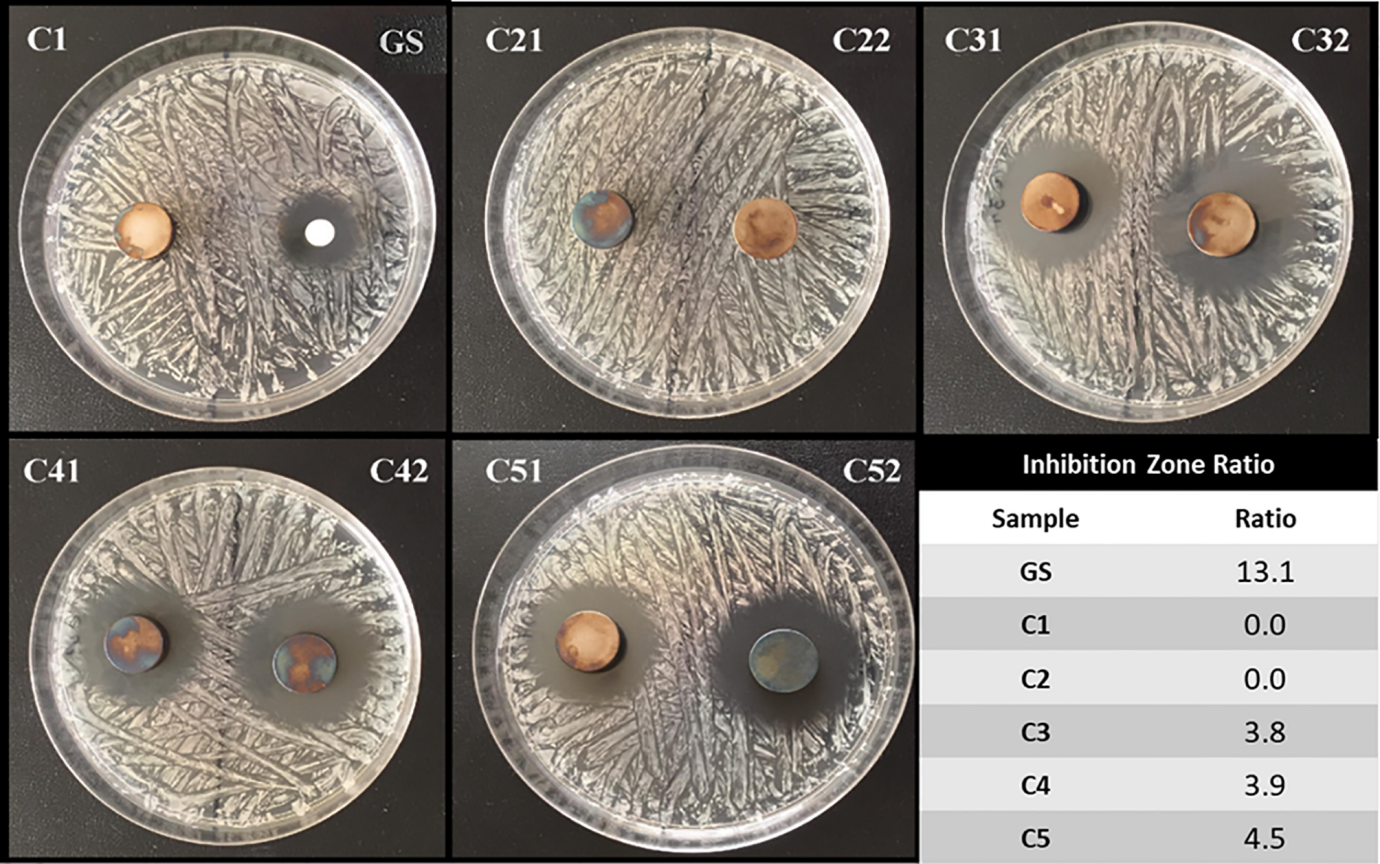
Inhibition zones of Ti6Al4V discs.

The inhibition zone test has been performed in order to evaluate the direct antibacterial effect of the produced coatings on *S. aureus* bacterial strain. **Figure 6** shows representative images of the tested conditions along with the calculated efficiency ratios. As show, the GS-loaded control was able to efficiently inhibit *S. aureus* growth, as expected. On the contrary, the C1 and C2 samples, both not containing GS, did not exert any effect on the treated bacteria. Interestingly, the C3, C4 and C5 conditions, all containing GS, were able to impeach bacterial growth around them, showing that the released gentamicin was still functional after release. Of interest, despite containing the same concentration of GS, the efficiency ratio for these conditions is lower than the one presented by control, thus suggesting a controlled release of GS from the samples.

**Figure 7:**
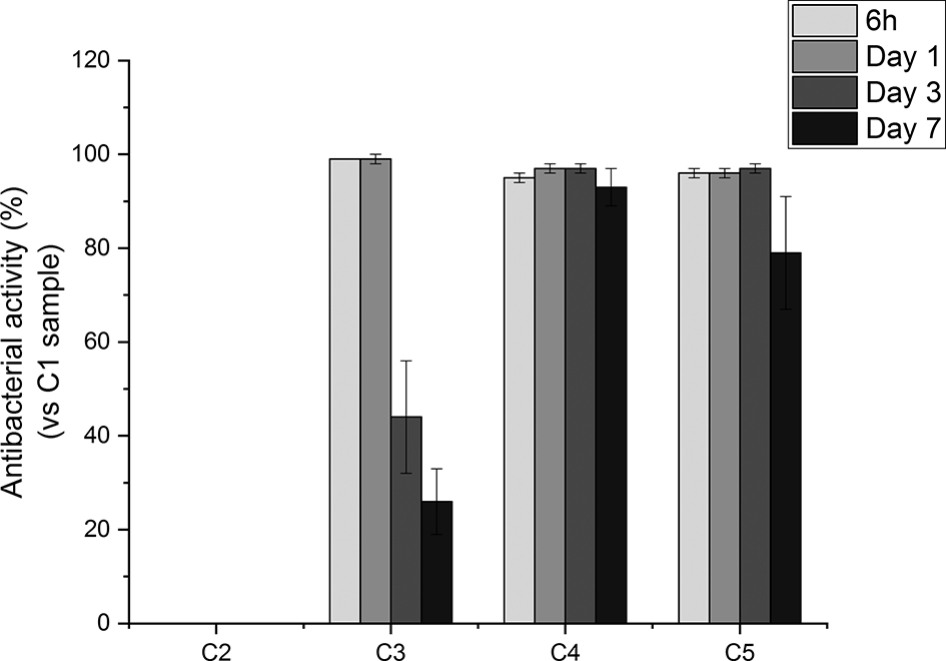
Percentage of antibacterial activity of Ti6Al4V-coated samples (i.e., C2, C3, C4, C5) with respect to uncoated Ti6Al4V surfaces (i.e. C1) against *S. aureus* 24 hours after inoculation.

To further evaluate the antibacterial effects exerted by the produced coatings, *in vitro* susceptibility tests were performed to evaluate the antibacterial activity over time of the released GS from agarose-coated Ti6Al4V samples compared to the uncoated ones. To relate the antibacterial activity of such coatings to that of pure GS, we evaluated the MIC of the antibiotic as well. Specifically, the minimum inhibitory concentration of GS against *S. aureus* resulted to be 1 μg/mL (see **SI -** **Figure S2**). This concentration is consistent with the values reported in the ISO norm, that is between 0.12 and 1 μg/mL (The European Committee on Antimicrobial Susceptibility Testing, Version 5.0, 2015, 2015). As shown in **Figure 7**, eluates collected from all the GS-loaded coatings inhibited bacterial growth. Indeed, the antibacterial activity of C3, C4 and C5 eluates collected after 6 h and 1 day was around 100%. Of note, eluates collected from agarose-coated samples (i.e., C2), displayed no antibacterial activity. In contrast with the C3 conditions, that showed a decrease in antibacterial effects with time, C4 and C5 samples showed a prolonged antibacterial effect. Indeed, C4 and C5 eluates collected after 3 days of incubation displayed a >99% antibacterial activity, while the antibacterial activity of C3 at the same time point was reduced to 44%. Furthermore, the eluate collected after 7 days still showed an antibacterial activity around 100%, while the effectiveness of the eluates collected from C3 samples dropped to 26%. Overall, such results suggest that TA may play a key role in slowing down the release of GS from coatings, thus helping to modulate the antibiotic release overtime.

## Discussion

Orthopedic implant-related infections can lead to delayed healing, implant loosening and even death. In order to eradicate the infection, implant debridement and removal are often required. Nowadays, implants with antibacterial effects are needed for the prevention and treatment of deep infection (Goodman et al., 2013). The presence of infection at the implantation site has adverse effects on the final surgical operation outcome, thereby delaying tissue healing and compromising the implant integration in the host. To this end, an antibiotic-loaded hydrogel coating study was carried out to increase the antibacterial activity of the Ti6Al4V implants used in the surgical treatment of spinal diseases. Ti6Al4V disc-shaped surfaces were coated with four different agarose-polyphenol based hydrogel formulations. Coating performance, antibacterial effect and cytotoxicity of discs were determined by surface characterization methods and *in vitro* tests. Surface activation has been carried out to create hydroxyl groups on the sample surfaces. Contact angle measurements shown how NaOH activation has been able to increase the hydrophilicity of the treated surfaces by forming hydroxyl groups on the surface for all concentrations tested, as expected based on literature (Kim et al., 2013). Moreover, the increase in NaOH concentration correlated with increased surface roughness (**Table 2**). However, high surface roughness is known in literature for being breeding ground for bacterial contamination, colonization, and biofilm formation (Ivanova et al., 2011; Han et al., 2016; Orapiriyakul et al., 2018). In fact, biofilm formation is higher on rough surface than on smooth ones (Han et al., 2016). In addition, Teughels et al. demonstrated that a surface roughness around 0.2 µm allows to decrease bacteria adhesion (Teughels et al., 2006). In this light, the optimum NaOH concentration, able to induce the formation of hydroxyl groups without changing the surface roughness, was determined at 2.5 M. Thus, for subsequent studies, surface activations were performed with 2.5 M NaOH. DOPA was next used as an intermediate layer to covalently graft the agarose hydrogel. In fact, covalent bonds between the hydrogels and the surfaces will increase the coating stability in the long term, as demonstrated by Campelo et al. (Campelo et al., 2017). According to XPS analyses (**Table 3**), this grafting method was effective on Ti6Al4V, as no Ti elements from the substrate were detected. Besides, the atomic percentages of C, O and N were close to those expected based on dopamine chemical structure. These results are corroborated by other studies, showing the formation of a polydopamine layer on Ti substrates (Kang et al., 2013; He et al., 2014; Wang et al., 2019). The DOPA-grafted surface exhibited similar values of contact angle and roughness when compared to that of the initial sandblasted alloy (**Table 2**). Despite a drastic increase in contact angle measurements of DOPA-grafted surface was noted, as compared to the highly hydrophilic NaOH-activated surfaces, still the DOPA-grafted surface remained hydrophilic. These observations, and similar contact angle values after DOPA grafting (~ 60°), have been previously described by Wang et al. on Ti samples (Wang et al., 2019), and by Campelo et al. on steel surfaces (Campelo et al., 2017). Agarose, a natural polymer commonly used for controlled drug release systems (Wang and Wu, 1997; Grolman et al., 2019; Salati et al., 2020), was chosen to obtain the antibiotic-loaded hydrogel for Ti6Al4V surfaces coating. Agarose displays several advantages such as its low cost, high availability, and non-cytotoxicity (Das and Pal, 2015). It is recognized that by controlling the agarose hydrogel porosity (Marras-Marquez et al., 2014; Khodadadi Yazdi et al., 2020), *via* its degree of crosslinking, the drug release can be modulated. Therefore, in order to have a better control on the antibiotic release kinetic and hydrogel cohesion and based on literature data, the effect of TA and CaCl_2_, were evaluated. The XPS surface characterization technique evidenced the agarose presence, by showing C and O percentages close to the one of pure agarose, regardless of the coating composition (**Table 3**). Furthermore, percentages of N associated to the previous dopamine layer became negligible, meaning that the agarose hydrogel was around 5 nm thick (XPS depth analysis) and homogeneously coated on DOPA sample. This was further corroborated by profilometry analysis, as the roughness remained close to the DOPA-grafted samples, between 0.55 μm and 0.65 μm (**Figure 3**). The highest roughness was observed for C4 sample (containing TA), that exhibited also the upmost contact angle value (~70°; **Figure 2**). This may be associated to the presence of the TA aromatic rings at the surface, as evidenced by the high-resolution C1s spectra (**Figure 1**). Despite these slight differences, the different agarose-based coatings, from C2 to C5, appeared homogeneous and exhibited a hydrophilic character. In addition, the agarose-based coatings have been shown to be stable under pseudo-physiological environment after 28 days (FTIR spectra in **Figure S1**), meaning that the agarose-based hydrogels are, as expected, grafted to the dopamine intermediate layer. There is substantial literature on agarose-based hydrogels but, to the best of our knowledge, only few articles are related to their grafting on surfaces. Besides, all of them require agarose chemical modification. For instance, Xu et al., have grafted agarose on Ti-DOPA surface by click chemistry, leading to a fully covered Ti surface (Xu et al., 2016b). Despite interesting results, as this surface allowed to decrease sea bacteria adhesion, the need of agarose and DOPA activations using complex chemical reactions and purification steps make this approach complex. The authors investigated a more friendly approach based on modified agarose by bromine mixed with TA at different ratios on Ti surfaces (Xu et al., 2017). The resulting coatings are more hydrophilic (< 40°) compared to the contact values obtained herein (~ 60°). However, their XPS results evidenced that the Ti surface is not fully covered as Ti is still detected. Also, they demonstrated that a higher TA ratio led to a better covering due to the polyphenol interaction with the substrate, allowing a better durability under ageing. Even though the agarose hydrogel composition did not significantly change the surface characteristics and rating stability, the effect of the coating composition on the release of GS did not exhibit the same behavior. Despite the burst release occurring in the first 6 hours for all conditions, the TA presence, C4 and C5, added to ensure long-term release of GS, appeared to delay the GS release, compared to C3 sample, condition without TA (**Figure 4**). This can be related to the phenolic groups of TA, well known to induce crosslinking with the surrounding molecules herein the agarose (Zhang et al., 2010; Ninan et al., 2016), with the added effect of UV irradiation (Behboodi-Sadabad et al., 2017). Indeed, it is recognized that higher degrees of crosslinks correlate with slower release times (Meng et al., 2014; Behboodi-Sadabad et al., 2017). After the burst release, C4 and C5 samples exhibited a slow antibiotic release over the next 7 days with an amount of GS released above the MIC value against *S. aureus* bacteria (1 µg/mL) (Naraharisetti et al., 2005). However, after 14 days of incubation, the GS release behavior was similar in samples containing TA and CaCl_2_ that was reduced below this MIC value. In clinic, the first 6 hours after implantation are considered as a “decisive period” for the development of implant-related infections (Hetrick and Schoenfisch, 2006). During this period, the implant is highly susceptible to bacterial colonization. Moreover, the infecting pathogens are passive during this period, representing an opportunity to kill them (Poelstra et al., 2002). Accordingly, Vasilev et al. suggested that an ideal antibiotic-release coating should provide a rapid release for 6 hours followed by a slower release (Vasilev et al., 2009). Cytotoxicity tests have been performed in order to evaluate if the herein formulated agarose-base delivery systems exerted any toxic effects on human cells. To do so, indirect cytotoxicity tests have been performed on Saos-2 cells. Saos-2 are an osteosarcoma-derived permanent cell line possessing osteoblastic features that have allowed, over the years, their use as a cell model for bone-related studies (Rodan et al., 1987; McQuillan et al., 1995; Saldaña et al., 2011; Prideaux et al., 2014). The results of the test showed how condition C1 and C2 did not exert any cytotoxic effect on the tested cells. This is in accordance with previous literature regarding the non-cytotoxicity of both DOPA coatings (Lee et al., 2014) and agarose hydrogel degradation products (Zhang et al., 2012; Pauly et al., 2017), and demonstrate the safety of the proposed surface modification approach. Regarding the GS-releasing C3 condition, once again no cytotoxic effects have been noted. GS is often chosen as an antibiotic thanks to its broad spectrum of efficiency. However, toxicity has been noted with the use of systemic administration. For this reason, the local and controlled release of GS has to be ensured in order to avoid negative secondary effects (Yang et al., 2018). In our experiments, the amount of released GS falls in the concentration window in which GS has been shown to not exert cytotoxic effects (Isefuku et al., 2003). However, cytotoxicity resulted being statistically increased in the presence of conditions C4. This condition contains TA as a crosslinking agent. TA has been shown, over years, antitumoral activity against several cancer cell lines (Nam et al., 2001; Karakurt and Adali, 2016; Zhang et al., 2018; Nagesh et al., 2020). As previously stated, despite being widely used for bone-related studies, the hereby used Saos-2 cell line derive from human osteosarcoma, a well-known type of bone cancer. Therefore, the increase of cytotoxicity recorded with the C4 samples may be attributed to the used cellular model. Interestingly, in presence of the C5 condition, the recorded cytotoxic effects on Saos-2 were similar to those observed for the C1, C2 and C3 conditions (**Figure 5**). Therefore, the addition of CaCl_2_ to the gel formulation was able to mask the negative effects noted in presence of TA. The beneficial effects of CaCl_2_ have been previously reported on Saos-2 cells (Cibor et al., 2017; Hou et al., 2020), explaining the obtained results. To evaluate the antibacterial activity of the formulated coatings, GS-containing samples have been tested against *S. aureus*, a Gram-positive bacterium that is the first cause of spinal implants-associated infections (Quaile, 2012). The ZOI test demonstrates the direct effect of the GS released by the tested samples. All of the GS-containing conditions (C3, C4 and C5) were able to induce bacteria inhibition in the area surrounding the samples, demonstrating the ability of the loaded GS to diffuse from the prepared hydrogel coatings, in accordance with literature (Moskowitz et al., 2010; Cibor et al., 2017). The susceptibility test shed more light on the antibacterial effects of the produced coatings. For instance, they demonstrated how the released GS, over time, was able to maintain its effectiveness once released. Moreover, the results highlighted the controlled release obtained with the addition of the crosslinking agents TA and CaCl_2_. In fact, the C3 condition started to lose effectiveness over time, in accordance with the lower amount of the released GS. As for conditions C4 and C5, their antibacterial effectiveness at longer time points (2 and 7 days) resulted significantly higher compared to C3 samples. Therefore, the increase in crosslinking can be directly associated with a slower release of GS, allowing to have high antibacterial activity over time (Campos et al., 2009).

## Conclusions

In the present study, Ti6Al4V disc surfaces were coated with GS-loaded agarose hydrogels and their antibacterial effect and the potential toxic effect on human cells were investigated. Homogeneous agarose-based antibacterial coatings were successfully obtained, as highlighted by the performed physicochemical characterizations, by means of simple experimental procedures relying on the use of safer, more friendly chemicals. The concentration of released GS from the antibiotic-loaded coatings was higher than the MIC of the antibiotic for up to 7 days, with a more pronounced controlled release effect in the presence of the crosslinking agents TA and CaCl_2_. Moreover, as shown by the antibacterial tests, the produced coating and the released GS were able to efficiently inhibit bacterial activity. Furthermore, the studied coating generally showed negligible cytotoxic effects towards the tested human cells. In this light, the hereby-presented results open the path for the further development of agarose-based antibacterial coatings for spinal implants modification. Still, the performances of the produced coatings in an *in vivo* environment should be further investigated, in terms of both antibacterial activity and osseointegration, in order to disclose their fully potential for therapeutic applications. Of note, the hereby-presented coating approach might also be translated to orthopedic applications and bone implants, making it a viable platform for addressing orthopedic-related infections.

## Data Availability Statement

The raw data supporting the conclusions of this article will be made available by the authors, without undue reservation.

## Author Contributions

HS, FY, and DM conceived the presented idea. HS, PC, FC, GC, FY, and DM conceived and planned the experiments. HS carried out the experiments with the help of PC, FC, FP and GC. HS, PC, FC, FP, and GC contributed to the interpretation of the results. HS wrote the manuscript with substantial help from PC and FC and in consultation with GC, FY and DM. FY and DM supervised the project. All authors contributed to the article and approved the submitted version.

## Funding

This research was partially supported by the Natural Science and Engineering Research Council of Canada (Discovery program, Strategic and INNOV-I2I, CUI projects), by the Scientific and Technological Research Council of Turkey (TUBITAK) (Project Number: 1059B141700535), and by Ege University BAP (Project Number:16FBE025).

## Conflict of Interest

The authors declare that the research was conducted in the absence of any commercial or financial relationships that could be construed as a potential conflict of interest.
