# Supplementary material for: A Novel Strategy to Coat Dopamine-Functionalized Titanium Surfaces With Agarose-Based Hydrogels for the Controlled Release of Gentamicin
Source: Front Cell Infect Microbiol. 2021 Jun 10;11:678081. doi: 10.3389/fcimb.2021.678081 (PMC8224171; doi:10.3389/fcimb.2021.678081)
Supplement: Supplementary file 1 [file Table_1.docx]

**SUPPORTING DATA**

**Coating stability over 28 days**

**
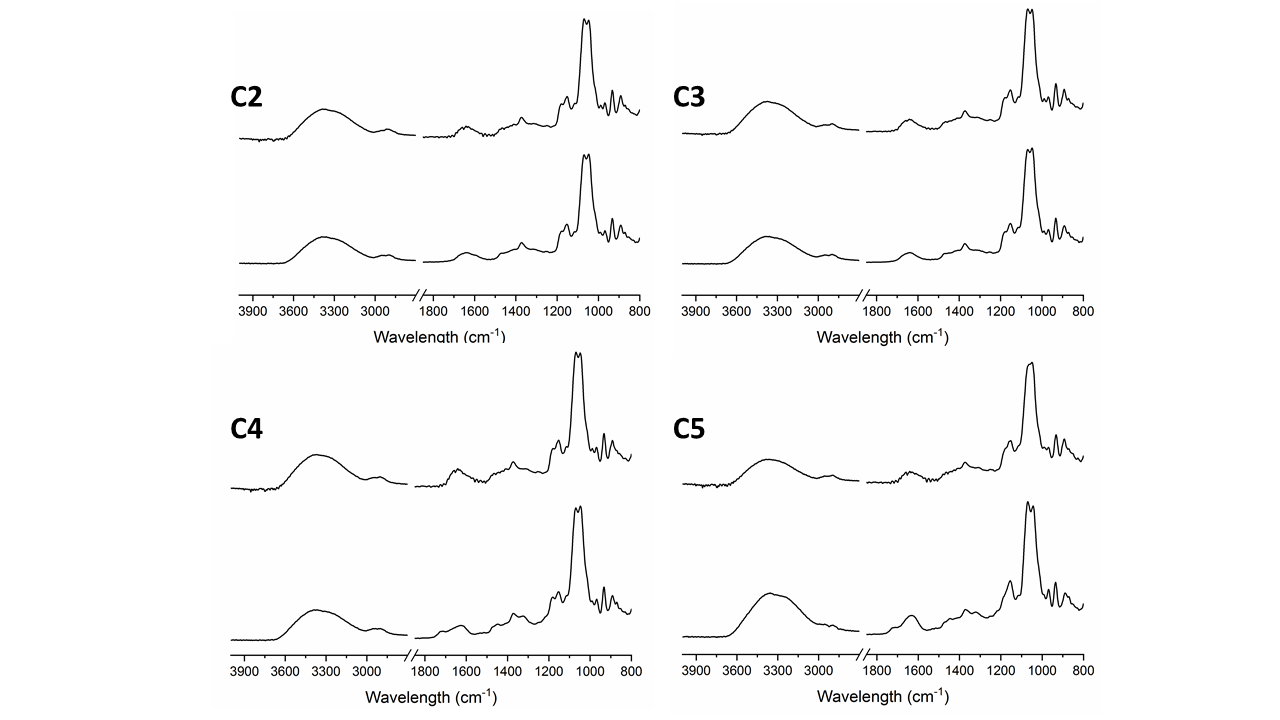
**

**Figure SI1.** FTIR Spectra of the different coatings before (spectra down) and after 28 days of GS release test (spectra up).

As shown in Figure SI1, whatever the coating composition, the FTIR spectra before and after 28 days release test remain unchanged. Indeed, the main characteristic peaks, of agarose, at 1046 cm^-1^ and 931 cm^-1^, are still visible. They belong to the C-O strain vibration and the 3,6-anhydrogalactose group in agarose, respectively. It means that the C2, C3, C4 and C5 coatings are stable over 28 days.

**MIC evaluation of gentamicin**

As shown in Figure S2, the minimum inhibitory concentration of gentamicin against *S. Aureus* strain resulted to be 1 μg/mL. This concentration is consistent with the values reported in the ISO norm, that is between 0.12 and 1 μg/mL.





**Figure S2** Determination of the MIC of gentamicin against *S. aureus.*
